# Predictors of attendance at the first follow-up and poor visual outcome after paediatric cataract surgery in Kinshasa for the years 2001–2021

**DOI:** 10.1186/s41182-025-00706-8

**Published:** 2025-02-26

**Authors:** Helene Schulz, Elena Rakuša, Stefanie Frech, Thomas Stahnke, Ngoy J. Kilangalanga, Rudolf F. Guthoff, Gabriele Doblhammer

**Affiliations:** 1 Department of Sociology and Demography, Chair of Empirical Social Research and Demography, University of Rostock, Ulmenstraße 69, 18057 Rostock, Germany; 2 German Center for Neurodegenerative Disease, Bonn, Germany; 3 Department of Ophthalmology, Rostock University Medical Center, Rostock, Germany; 4 Eye Department, St. Joseph Hospital/CFOAC, Kinshasa, Democratic Republic of the Congo; 5 Eye Unit, Université Catholique du Graben, Butembo, Democratic Republic of the Congo

**Keywords:** Paediatric cataract surgery, Follow-up, Visual acuity outcome

## Abstract

**Background:**

About 90% of blind children come from low- and middle-income countries. The main cause of childhood blindness is cataract. Cataract surgery can improve vision, but regular follow-up is necessary. Low attendance at follow-up is a medical and statistical challenge, as missing information can lead to biased results. Two research questions arise: what social factors influence attendance at first follow-up? What social factors influence a poor outcome of visual acuity at first follow-up?

**Methods:**

An observational cohort study was conducted, and the total base population was analysed. The study includes children who received cataract surgery at Saint Joseph Hospital (Kinshasa, Democratic Republic of the Congo) from 2001 to 2021. Cox regression was used to examine attendance at the first follow-up (*n* = 1100 operated eyes), ordinal logistic regression to analyse visual acuity at the first follow-up (*n* = 699 operated eyes), both clustered by patient identification number. Due to the high number of missing values, multiple imputation was performed as a sensitivity analysis.

**Results:**

Female sex, young age, very good visual acuity after surgery, and disease in both eyes led to lower attendance at follow-up. Poor visual acuity outcome at follow-up was associated with female sex, young age, poor financial situation, blindness after surgery and nystagmus.

**Conclusions:**

As regular follow-up is crucial for an improved visual acuity outcome, it is recommended that special attention needs to be paid to the groups identified in our analysis to better integrate them into follow-up. In particular, the higher risk of poor outcome in younger children is surprising and requires further analysis.

**Supplementary Information:**

The online version contains supplementary material available at 10.1186/s41182-025-00706-8.

## Introduction

In 2020, the World Health Organization (WHO) estimated that there were 43.3 million blind people in the world [1] among them ~ 1.4 million blind children [2]. About 90% of children affected by blindness are from low- and middle-income countries, of which 72% are from Asia and 18% from Africa [3]. The prevalence of blindness ranges from about 3–4/10,000 children in economically developed countries to 12–15/10,000 in the poorest countries [4]. Childhood blindness has profound and far-reaching personal and economic consequences. Blind children face a lifetime of blindness that limits their opportunities for education, employment and income. Early onset blindness also leads to delays in motor, language, social and cognitive development [5, 6].

One of the leading causes of childhood blindness is cataract [2], which is also the focus of many programmes implementing the goals of "Vision 2020—The Right to Sight", a global initiative to eliminate avoidable blindness [7]. Cataract surgery can correct visual acuity and restore sight [8]. However, African countries have particularly low levels of access to and coverage of basic health services. The establishment of tertiary facilities in African countries has been a first step in improving medical care for children with cataract [8]. This includes the Centre for Paediatric Ophthalmology at the Saint-Joseph Hospital in Kinshasa (Democratic Republic of the Congo), which has been collaborating with the University Medical Centre Rostock (Germany) since 2001 and whose clinical data form the basis of the study.

In addition to high quality and timely surgery, postoperative follow-up is critical for optimal visual outcomes. Studies of paediatric cataract surgery report low rates of routine follow-up in low- and middle-income countries, with ~ 20–40% of patients missing information on postoperative visual acuity [9–14].

Factors associated with low attendance include female sex [11], long distance from the hospital [11, 15], delay in presentation for surgery [11], high cost [13, 16, 17], low socioeconomic status [11], older child age or adolescence [13], and low maternal education level [11, 13].

Concerning visual acuity outcome in the first follow-up, early presentation and treatment is important. Infants and young children have better outcomes than older children [18–20]. However, a common problem in middle- and low-income countries is that children are delayed in getting to appropriate health care facilities, which negatively affects outcomes [21]. Generally, sex does not have an effect [22], with the exception of a study in Mexico where female children had worse outcomes [15].

Similarly, preoperative blindness and preoperative nystagmus (wobbling eyes) are associated with poor final visual acuity outcomes in children with bilateral cataract [14, 20, 23, 24]. However, bilateral cataract (both eyes affected) was associated with a better outcome than unilateral cataract (only one eye affected) [20, 25].

The aim of this paper is to analyse the social factors that influence participation and visual acuity at the first follow-up examination. There are few studies on the factors influencing non-attendance at follow-up after paediatric cataract surgery in low- and middle-income countries. We pay special attention to sex, age, family financial situation and hypothesise that they have an influence on attendance at the first follow-up such that older and female children from low-income families have lower attendance. We also suspect that higher age of the children may have a negative influence on the visual outcome, while for family income and sex there should be no differences in visual acuity. Furthermore, we assume that the medical pre-conditions nystagmus and laterality affect the outcome at the first follow-up such that those with nystagmus and unilateral cataract will have a worse outcome.

## Materials and methods

### Data

**Fig. 1:**
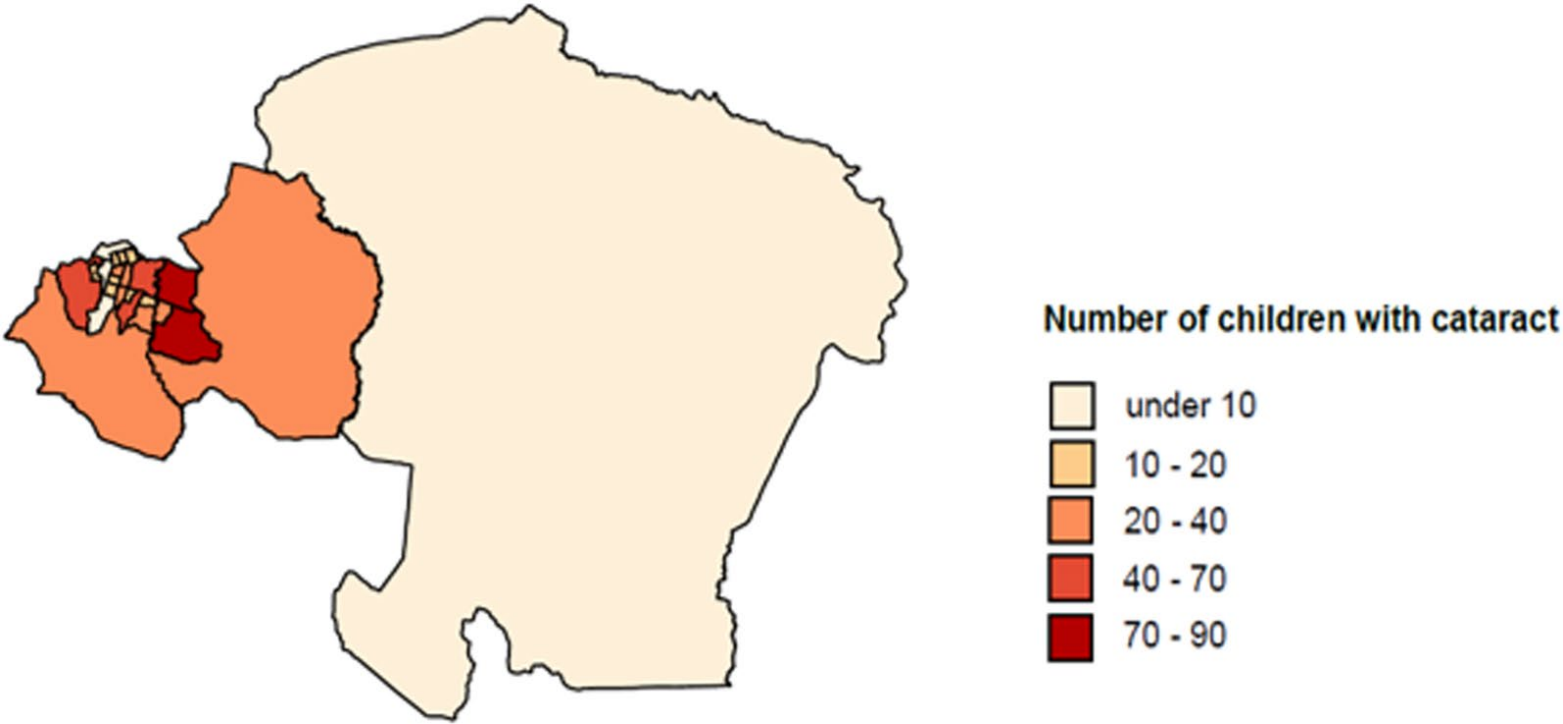
Number of children with cataract in the districts of Kinshasa, map visualization. Source: Saint Joseph Hospital (2001–2021)

The study employed a cohort observational design, with the entire base population being analysed. In a Christoffel Blind Mission (CBM) community-based rehabilitation programme in Kinshasa, children's eye conditions requiring treatment were identified by trained community volunteers and a referral was made to Saint Joseph Hospital [8], but also self-initiated hospital treatments were possible. The CBM screening programme covers a catchment area of 16 health zones in Kinshasa [8]. The distribution of children undergoing cataract surgery varies by district (Fig. 1). During diagnosis and treatment, socio-demographic, pre-operative, surgical and post-operative characteristics were recorded in a database. At 20 years, the study period covers an exceptionally long time with a large number of procedures. The average number of eyes operated on per year was 55, with the lowest number of operations in 2018 (18 eyes) and the highest in 2020 (117 eyes).

**Fig. 2:**
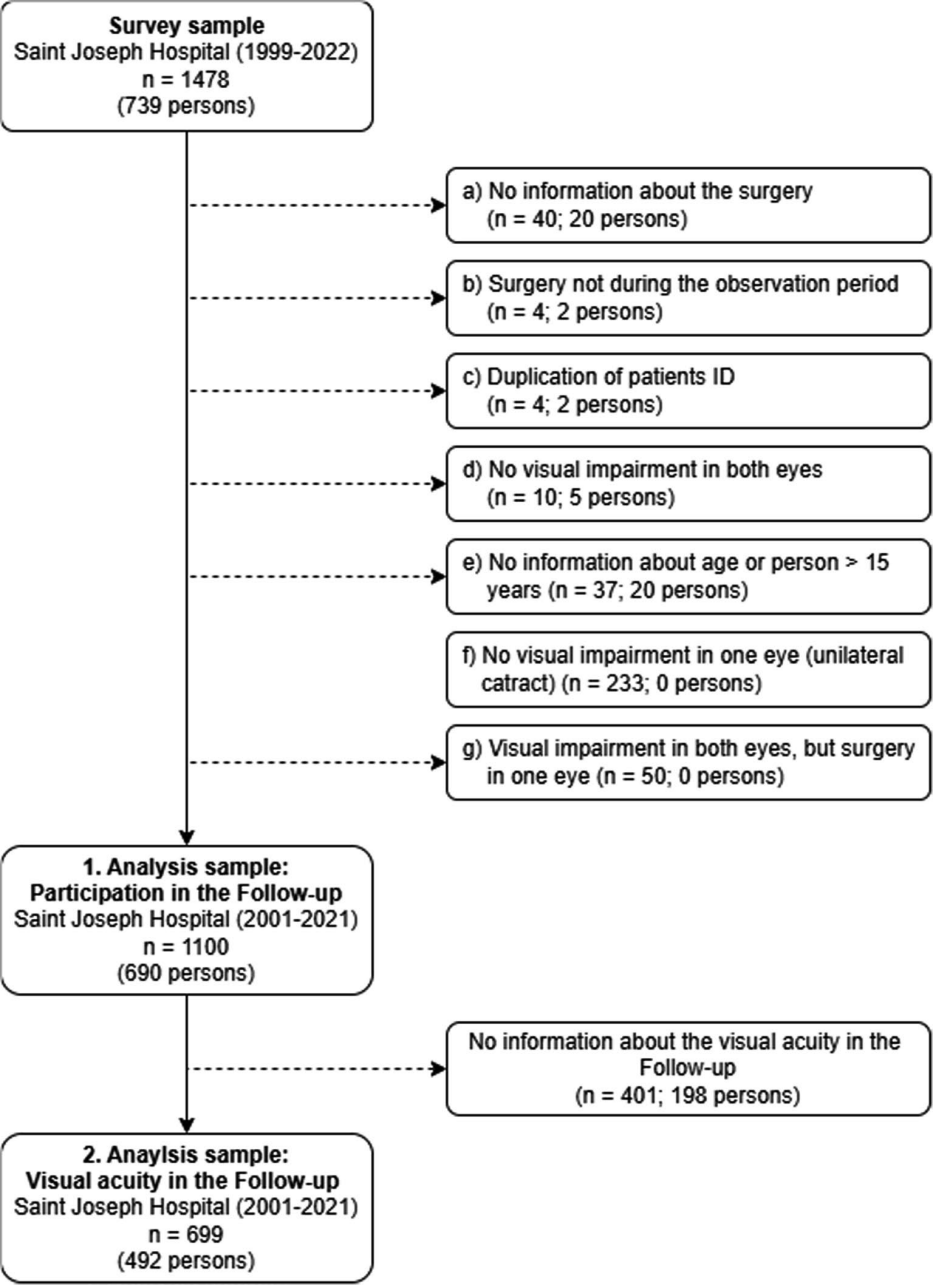
Analysis sample. Source: Saint Joseph Hospital 2001–2021

In the period from September 1999 to March 2022, 739 children with cataract were presented at Saint Joseph Hospital, corresponding to *n* = 1478 examined eyes. Figure 2 shows our exclusion criteria (a–g): Only eyes for which cataract surgery could be reliably determined during the period 2001–2021 were included in our study. Accordingly, 40 observations were excluded where no information on the surgery was documented (a) and 4 observations where the surgery was outside the observation period (b). Five children had no cataract, therefore their eyes were excluded from analysis (*n* = 10) (c). Observations with duplicate patient identification numbers (*n* = 4) (d), cases without age information and cases older than 15 years were excluded (*n* = 37) (e). For eyes that underwent multiple surgeries due to complications, the first postoperative finding was included in the analysis for better comparability. In case of a unilateral cataract, eyes without visual impairments were excluded (*n* = 203) (f) and eyes affected by cataract that had not yet been operated on were also excluded (*n* = 50) (g). As the eyes of children with bilateral cataracts were sometimes operated on at different times (up to several months), we analyse participation by looking at the first follow-up visit at 3 weeks separately for each eye. Consequently, the analysis of participation in the initial follow-up examination was based on the data set comprising 1100 operated eyes in 690 individuals (1. Analysis sample). Among them information on visual acuity at first follow-up was missing for n = 401 eyes and 198 persons, resulting in *n* = 699 eyes and 492 persons for the analysis of visual acuity at the first follow-up (2. Analysis sample).

### Outcome

Participation in the first follow-up after 3 weeks (± 14 days as a variable time window) was defined as information on the date of the first follow-up. Individuals who had no information about the date of the follow-up or who attended the follow-up outside the defined period were counted as non-participants. Visual acuity at the first scheduled follow-up was assessed separately for the right and left eye, with categorisation based on the International Classification of the World Health Organisation (WHO): "No impairment: Visual acuity ≥ 6/18", "Moderate impairment: Visual acuity between 6/18 and 6/60", "Severe impairment: Visual acuity from 6/60 to 3/60", "Follow light/ Follow object", "Blind: Visual acuity < 3/60" [26].

### Explanatory variables

We explored the effects of sex: female or male, age at time of surgery centred on the median age, family financial situation: destitute/poor (< 80$ per month), low income (80–150$ per month) or regular income (150 + $ per month).

### Control variables

Medical control variables were laterality (unilateral; bilateral), and nystagmus (present; absent) before surgery and visual acuity immediately before and after surgery, defined similarly to visual acuity at first follow-up (see section Outcome). In the analysis of visual acuity in the first follow-up, the categories "Blind: < 3/60" and "Follow light or object" (preoperative) were combined and the categories "Severe visual impairment: 6/60 to 3/60", "Blind" and "Follow light or object" (after surgery).

### Analysis strategy and statistical methods

Cox regression was used for the analysis of participation at the first follow-up and ordinal logistic regression for the analysis of poor visual acuity at the first follow-up. Since in both analyses our observations were eyes that underwent surgery, we clustered the variances by patient identification number. Due to the high number of missing values for visual acuity at the first follow-up, a sensitivity analysis was performed using multiple imputations of visual acuity as well as of the explanatory variables (visual acuity immediately before surgery, visual acuity after the surgery, family financial situation) combining them into one analysis based on Rubin [27]. All analyses were performed using STATA 12.1.

The data collection documenting the cataract surgeries complied with local legislation and the tenets of the Declaration of Helsinki. If additional procedures were proposed, parents were informed and asked to sign a consent form. Our study involved only retrospective, anonymised data and fell outside the scope of the Declaration of Helsinki and did not require ethical review. This was certified by the ethical review board of the Medical University of Rostock.

## Results

### Descriptive results

There was a first follow-up examination for 71% of the eyes. Of these, 16.0% were blind, 7.4% could follow light or objects, 37.6% were severely visually impaired, 29.8% were moderately visually impaired and 9.2% had no visual impairment.

The sex distribution was 62.3% (male) to 37.7% (female). The mean age of the children at the time of surgery was 5.7 years, with a range of 0 to 15 years. 7.6% came from destitute/poor families (< 80$ per month), 71.8% with low income (80–150$ per month) and 19.1% with regular income (150$ + per month).

A large proportion (63.6%) of the 1100 eyes examined before surgery (Table 1) were blind with visual acuity < 3/60, while a further 19.8% had severe visual impairment (visual acuity < 6/60 to 3/6). A total of 8.3% of the eyes were found to be capable of following light and objects. Moderate impairment was seen in 6.9% of observations with visual acuity of 6/18 to 6/60. Significantly fewer eyes were blind immediately after surgery (9.2%), but 53.3% of eyes still had severe impairment, 1.6% to be capable of following light or objects, 14.7% had moderate impairment and 2.0% had no impairment. At the first follow-up, 10.2% of eyes were blind, 4.7% could follow light or objects, 23.9% had severe impairment, 18.9% had moderate impairment and 5.8% had no impairment. Nystagmus was observed in 22.4% of eyes.

**Table 1:** Demographic and clinical information

Variable	Value	1. Analyse sample		2. Analyse sample	
		*N*	%	*N*	%
Participation	No	319	29.00		
	Yes	781	71.00		
Visual acuity	Not impaired	64	5.81	64	9.16
(follow-up)	Visual impairment	208	18.91	208	29.76
	Severe visual impairment	263	23.91	263	37.63
	Follow light or object	52	4.73	52	7.44
	Blind	112	10.18	112	16.02
	Missing	401	36.45	0	0.00
Sex	Male	685	62.27	457	65.38
	Female	415	37.73	242	34.62
Age	mean	5.69		6.23	
Financial situation	Destitute/poor	83	7.55	46	6.58
	Low income	790	71.82	517	73.96
	Regular income	210	19.09	124	17.74
	Missing	17	1.55	12	1.72
Visual acuity	Not impaired	0	0.00	0	0.00
(preoperative)	Visual impairment	76	6.91	58	8.30
	Severe visual impairment	218	19.82	140	20.03
	Blind	700	63.64	491	70.24
	Follow light or object	91	8.27		
	Missing	15	1.36	10	1.43
Visual acuity	Not impaired	22	2.00	11	1.95
(after surgery)	Visual impairment	162	14.73	117	16.55
	Severe visual impairment	586	53.27	463	66.24
	Blind	102	9.27		
	Follow light or object	17	1.55		
	Missing	211	19.18	108	15.45
Laterality	Unilateral	226	20.55	178	25.46
	Bilateral	874	79.45	521	74.54
Nystagmus	No	854	77.64	553	79.11
	Yes	246	22.36	146	20.89
Total		1100	100.00	699	100.00

1. Analyse sample: participation in the first follow-up; 2. Analyse sample: visual acuity in the follow-up; *n* = number of eyes

Source: Saint Joseph Hospital 2001–2021

Analysing the missing value (Supplementary Table S1), we observed a pattern consistent with a missing at random (MAR) process thus justifying imputation. The parameter estimates are summarised according to the Rubin approach [27].

### Multivariate analysis of participation in the first follow-up

The gross effects (models 1a–1c) showed a higher risk of follow-up for older children and for children from poor families (Table 2). In the full model 2, there was a borderline significance for sex, with girl exhibiting a 16% lower risk of attending follow-up compared to boys (Table 2). An age gradient is evident: The risk of attending the first follow-up increased 1.03-fold with each year of age. There was a borderline significance for visual acuity before surgery, with blind children and children with severe visual impairment having an ~ 28% lower risk of participating in the first follow-up. Compared to people with unilateral cataract, people with bilateral cataract had a 29% lower risk of attending the follow-up. No statistically significant differences existed for visual acuity (after surgery), financial situation and nystagmus.

**Table 2:** Hazard ratio (HR) of participation in the first follow-up

Variable	Value	Model 1a–c		Model 2	
		HR	*p*-value	HR	*p*-value
Sex	Male	1		1	
	Female	0.87	0.107	0.84	0.052
Age	Centred on median	1.03	0.006	1.03	0.013
Financial situation	Destitute/poor	1.5	0.025	1.05	0.812
	Low income	1		1	
	Regular income	1.10	0.425	1.04	0.718
	Missing	1.58	0.221	1.33	0.417
Visual acuity	Moderate visual impairment			1	
(preoperative)	Severe visual impairment			0.72	0.055
	Blind			0.73	0.061
	Follow light or object			1.35	0.220
	Missing			0.90	0.812
Visual acuity	Not impaired			0.57	0.104
(after surgery)	Moderate visual impairment			1.24	0.206
	Severe visual impairment			0.99	0.965
	Blind			1	
	Follow light or object			1.89	0.154
	Missing			1.40	0.081
Laterality	Unilateral			1	
	Bilateral			0.71	0.001
Nystagmus	No			1	
	Yes			1.02	0.827

Model 1a: sex only; Model 1b: age only; Model 1c; financial situation only

Source: Saint Joseph Hospital 2001–2021

### Multivariate analysis of poor visual acuity after cataract surgery in the first follow-up

The gross effects (models 1a–1c) showed a higher risk of visual impairment for females and a declining risk of visual impairment with age (Table 3). After adjustment for all covariates, there was a borderline significant effect of sex with female children having a 1.38-fold higher risk of poor visual acuity (Table 3; model 2). There was an age gradient: The risk of poor visual acuity decreases by 12% with each year of age. We did not find a significant effect of financial situation of the family. People who were blind or could only light or objects before surgery had a 2.29-fold higher risk of visual impairment than people with moderate impairment. Compared to people with moderate impairment immediately after surgery, people with no postoperative impairment had an 87% lower risk of visual impairment at the follow-up, while people with severe postoperative impairment, who were blind or could only follow light or objects, had a 2.47-fold higher risk of visual impairment. Nystagmus was associated with a 2.04-fold higher risk of visual impairment. No statistically significant differences existed for laterality.

**Table 3:** Odds ratio (OR) of visual acuity in the first follow-up

Variable	Value	Model 1a–c		Model 2	
		OR	*p*-value	OR	*p*-value
Sex	Male	1		1	
	Female	1.55	0.012	1.38	0.057
Age	Centred on median	0.86	< 0.001	0.88	< 0.001
Financial situation	Destitute/poor	2.18	0.236	2.23	0.070
	Low income	1		1	
	Regular income	0.10	0.999	0.91	0.656
	Missing	1.74	0.496	0.68	0.683
Visual acuity	Moderate visual impairment			1	
(preoperative)	Severe visual impairment			1.49	0.224
	Blind/follow light or object			2.29	0.010
	Missing			9.61	0.017
Visual acuity	Not impaired			0.13	0.013
(after surgery)	Moderate visual impairment			1	
	Severe visual impairment/blind/follow light or object			2.47	< 0.001
	Missing			8.29	< 0.001
Laterality	Unilateral			1	
	Bilateral			0.84	0.408
Nystagmus	No			1	
	Yes			2.04	0.001
Cut 1				− 1.22	
Cut 2				1.06	
Cut 3				3.05	
Cut 4				4.71	
N		699		699	
Log-likelihood				− 836.0804	
LR-Wert				< 0.001	
Pseudo-*R*^2^				0.1659	

Model 1a: sex only; Model 1b: age only; Model 1c; financial situation only

Source: Saint Joseph Hospital 2001–2021

Using multiple imputation estimates converged (Supplementary Figure S1) and results remained almost unchanged (Supplementary Table S2) with two important exceptions: The sex difference now became significant with females having a higher risk of visual impairment (OR = 1.52; *p*-value = 0.008), which was also true for the lowest income group (OR = 3.92; *p*-value = 0.002).

## Discussion

Based on clinical data from Saint Joseph Hospital, 71% of children attended their cataract surgery follow-up. Boys and older children were more likely to attend the follow-up visit, but we found no effect of financial situation. Poor visual acuity at the first follow-up was associated with younger age, female sex, and poor financial situation.

### Participation in the first follow-up

The percentage of children lost to follow-up is similar to other studies from low- and middle-income countries, ranging from 20 to 40% [9, 11, 12]. In line with Eriksen et al. [11], girls were less likely to be taken to the follow-up than boys. It is likely that these sex differences are due to the social role of the male sex in the community, with treatment and a good visual outcome being considered more important for boys to enable them to attend school [28]. Sex differences are already evident in the uptake of cataract surgery at Saint Joseph's Hospital, where there are two boys for every girl. Studies in low- and middle-income countries show similar results [9, 11, 13, 18]. In this context, it is particularly important to encourage girls with cataracts to undergo cataract surgery and follow-up examinations. This will enable them to access education and reduce their dependence on family and community members.

While Gogate et al. [13] confirm poor follow-up especially for older children (age ≥ 11 years), our results show a reduced risk of participation for younger children. We suggest that for younger children who are not yet in school, good vision is seen as less important. Qualitative studies have shown that when parents seek eye care, they consider improved vision to be an important factor in their children's school performance [29, 30]. However, further qualitative research is needed to find out why parents do not bring their children back for follow-up and to what extent the age of the children is important in the decision. The Democratic Republic of the Congo is a low-income country, with ~ 60% of people living on less than USD 2.15 per day in 2022 [31]. Based on data from Saint Joseph Hospital, ~ 72% of people have a monthly income of USD 80–150. A more precise categorisation of the financial situation was not possible with the data. In terms of the financial situation, we did not find any effect on participation in the follow-up. We expect the community-based rehabilitation (CBR) programme to have a positive impact, covering surgery costs and transport costs. Other studies have demonstrated that the cost of surgery is a deterrent to follow-up [13, 15]. However, we cannot exclude a data problem.

Children who had no visual impairment after surgery were less likely to return for follow-up, as were children with bilateral cataracts. Qualitative interviews showed that many parents were unaware of the importance of follow-up and did not consider it necessary to return their children to hospital if the visual outcome had improved [30]. According to Yorston et al. [9], children with immature cataracts who had relatively good vision preoperatively were more likely to attend follow-up, suggesting greater parental involvement. In line with the findings of Yorston et al. [9], we found a lower risk of attendance in children with bilateral cataracts. We assume that parents who bring their child with unilateral cataract to surgery are also more likely to try to attend follow-up and to be well informed about the treatment process.

### Visual acuity in the first follow-up

The proportion of blind children was significantly reduced from 62% before surgery to 16% at the first follow-up after 3 weeks. About 9% of the operated eyes achieved good visual acuity (6/18 or better) and about 30% achieved moderate visual acuity (6/18 to 6/60). In 7% of the observations it was not possible to determine a specific visual acuity, but these may follow light or objects.

Contrary to our hypothesis, the results show a higher risk of poor visual outcome at the first follow-up for girls. This is consistent with the findings in Mexico [15], but differs from other countries where no sex effect was found [22].

We found a lower risk of poor visual outcome in older children. This effect is different from other studies that predicted a better visual outcome in younger children [18–20]. These studies differ with regard to age groups and the partly separate evaluation of congenital (bilateral) and traumatic (unilateral) cataract. We only controlled for laterality and found no effect. Measuring visual acuity is particularly difficult in young children because no Snellen test is possible. Measuring visual acuity by assessing whether the child follows objects may lead to inaccuracies in the interpretation of the results. The age information is also partly based on estimates by the parents, which can also lead to inaccuracies.

Controlled for sex, age and medical precondition, financial situation does not affect the risk of participation in the follow-up, but is associated with worse medical outcomes in the sensitivity analysis using multiple imputation. Medical outcome studies do not usually control for financial situation, which makes comparisons difficult. Financial aspects play an important role in the treatment process for families [13, 16, 30], but the CBR programme covers direct costs and indirect costs such as transport. It is possible that the effect of financial situation on poor visual outcome is due to different living conditions in the families and less opportunity for home eye care after surgery. To understand the influence of financial situation on visual outcome after surgery, the extent to which living conditions differ by financial situation and how this affects visual outcome needs to be investigated in more detail.

Preoperative blindness, blindness and severe visual impairment immediately after surgery, and preoperative nystagmus were predictors of poor postoperative visual outcome; other outcome studies from low- and middle-income countries including children under 15 years of age with bilateral or unilateral cataract found the same results [14, 20, 23–25].

When comparing the results with previous studies [20, 25, 32], it is important to note that the follow-up times in these studies vary widely. To increase the validity within our study, we focused on the first follow-up after 3 weeks; other studies refer to the last follow-up, which can range from a few days to several years [20, 25, 32]. By clustering the data by patient identification number, we included both eyes in our analyses, not just the better eye [25, 32]. In addition, we imputed missing values when exploring visual acuity and this mainly influenced the results for sex and financial situation in terms of significance. This was important because visual acuity results were not available at the first follow-up for 36% of the observations.

### Strength

Our study has a series of strengths. Data availability is limited in low- and middle-income countries. The Saint Joseph's Hospital database offers a long study period of 20 years with follow-up of children with cataract-related visual impairment who have undergone surgery.

In addition to medical variables, social variables are also recorded, expanding the possibilities for analysis. Through the Community Based Rehabilitation (CBR) programme, which covers treatment and travel costs, there is no social bias in participation in the follow-up.

### Limitations

The study also has a number of limitations. Low follow-up rates in low- and middle-income countries lead to a high number of missing values, which may affect the assessment and interpretation of the results. Also in our study, 36% of the observations had no vision results at follow-up, but we used multiple imputation. We could not include all variables of interest (cause of cataract, mother's education, father's education, number of siblings) present in the dataset in our analyses because they had too many missing values (80%) or inconsistent coding.

## Conclusion

As a first step, we identified factors that have a positive influence on follow-up after cataract surgery in children. In the second part of our study, we analysed factors that influence visual outcome after surgery. As regular follow-up is crucial for a good visual outcome in cataract surgery, special attention should be given to girls and young children in family counselling to better integrate them into follow-up. Qualitative interviews remain important to better understand family dynamics and individual fears of treatment and follow-up, and to develop possible strategies to increase participation. Further research is needed to understand why the different financial circumstances of families affect the outcome of surgery and to develop recommendations for action where appropriate.

## Supplementary Information

Supplementary Material 1: Table S1: Missing Data Pattern. Figure S1: Convergence of means Source: Saint Joseph Hospital (2001–2021). Table S2: Odds ratio (OR) of visual acuity in the first follow-up, multiple imputation.

## Data Availability

The data that support the findings of this study are not openly available due to reasons of sensitivity and are available from the corresponding author upon reasonable request. The data used was provided by St Joseph Hospital (Kinshasa, DRC).

## References

[1] Bourne R, Steinmetz JD, Flaxman S, Briant PS, Taylor HR. Resnikoff Sea. Trends in prevalence of blindness and distance and near vision impairment over 30 years: an analysis for the Global Burden of Disease Study. Lancet Glob Health. 2021;9(2):e130–43. 33275950 10.1016/S2214-109X(20)30425-3 PMC7820390

[2] Solebo AL, Teoh L, Rahi J. Epidemiology of blindness in children. Arch Dis Child. 2017;102(9):853–7. 28465303 10.1136/archdischild-2016-310532

[3] Naipal S, Rampersad N. A review of visual impairment. Afr Vis Eye Health. 2018;77(1).

[4] Gogate P, Gilbert C. Blindness in children: a worldwide perspective. Community Eye Health. 2007;20(62):32–3. 17612696 PMC1906926

[5] Amiebenomo OM, Achugwo DC, Abah I. Parental knowledge and attitude to children’s eye care services. Nig J Paed. 2016;43(3):215.

[6] Welp A, Woodbury RB, McCoy MA, Teutsch SM, editors. Making eye health a population health imperative: vision for tomorrow. Washington (DC); 2016. 27656731

[7] World Health Organization. World report on vision. Geneva: WHO; 2019.

[8] Kilangalanga JN, Stahnke T, Moanda A, Makwanga E, Hopkins A, Guthoff RF. Role of a community-based program for identification and referral of pediatric cataract patients in Kinshasa, Democratic Republic of the Congo. Middle East Afr J Ophthalmol. 2019;26(2):83–8. 31543665 10.4103/meajo.MEAJO_273_18 PMC6737789

[9] Yorston D, Wood M, Foster A. Results of cataract surgery in young children in east Africa. Br J Ophthalmol. 2001;85(3):267–71. 11222328 10.1136/bjo.85.3.267 PMC1723882

[10] Limburg H, Foster A, Gilbert C, Johnson GJ, Kyndt M, Myatt M. Routine monitoring of visual outcome of cataract surgery. Part 2: Results from eight study centres. Br J Ophthalmol. 2005;89(1):50–2. 15615746 10.1136/bjo.2004.045369 PMC1772465

[11] Eriksen JR, Bronsard A, Mosha M, Carmichael D, Hall A, Courtright P. Predictors of poor follow-up in children that had cataract surgery. Ophthalmic Epidemiol. 2006;13(4):237–43. 16877282 10.1080/09286580600672213

[12] Rai SKC, Thapa H, Kandel RP, Ishaq M, Bassett K. Clinical and cost impact of a pediatric cataract follow-up program in western Nepal and adjacent northern Indian States. J AAPOS. 2014;18(1):67–70. 24568986 10.1016/j.jaapos.2013.09.008

[13] Gogate P, Patil S, Kulkarni A, Mahadik A, Tamboli R, Mane R, et al. Barriers to follow-up for pediatric cataract surgery in Maharashtra, India: how regular follow-up is important for good outcome. The Miraj Pediatric Cataract Study II. Indian J Ophthalmol. 2014;62(3):327–32. 24008794 10.4103/0301-4738.116465 PMC4061672

[14] Mndeme FG, Mmbaga BT, Msina M, Mwende J, Vaitha SJ, Kim MJ, et al. Presentation, surgery and 1-year outcomes of childhood cataract surgery in Tanzania. Br J Ophthalmol. 2021;105(3):334–40. 32522793 10.1136/bjophthalmol-2020-316042 PMC7907562

[15] Congdon NG, Ruiz S, Suzuki M, Herrera V. Determinants of pediatric cataract program outcomes and follow-up in a large series in Mexico. J Cataract Refract Surg. 2007;33(10):1775–80. 17889776 10.1016/j.jcrs.2007.06.025

[16] Huang G, Crooms R, Chen Q, Congdon N, He M. Compliance with follow-up after cataract surgery in rural China. Ophthalmic Epidemiol. 2012;19(2):67–73. 22448612 10.3109/09286586.2011.628777

[17] Bright T, Felix L, Kuper H, Polack S. Systematic review of strategies to increase access to health services among children over five in low- and middle-income countries. Trop Med Int Health. 2018;23(5):476–507. 29473273 10.1111/tmi.13044

[18] Adams C, Alex AA, Trivedi RH, Wilson ME. Outcomes of bilateral cataract surgery in children 2–7 years of age: a comparison to surgery in toddlers and infants. J AAPOS. 2022;26(3):133.e1-133.e6. 35577020 10.1016/j.jaapos.2022.02.011

[19] Chan WH, Biswas S, Ashworth JL, Lloyd IC. Congenital and infantile cataract: aetiology and management. Eur J Pediatr. 2012;171(4):625–30. 22383071 10.1007/s00431-012-1700-1

[20] Tomkins O, Ben-Zion I, Moore DB, Helveston EE. Outcomes of pediatric cataract surgery at a tertiary care center in rural southern Ethiopia. Arch Ophthalmol. 2011;129(10):1293–7. 21987671 10.1001/archophthalmol.2011.268

[21] Mailu EW, Virendrakumar B, Bechange S, Jolley E, Schmidt E. Factors associated with the uptake of cataract surgery and interventions to improve uptake in low- and middle-income countries: a systematic review. PLoS ONE. 2020;15(7): e0235699. 32645065 10.1371/journal.pone.0235699 PMC7347115

[22] Negretti GS, Ayoub T, Ahmed S, Deb R, Majumder U, Jewel J, et al. Cataract surgery outcomes in Bangladeshi children. Ophthalmology. 2015;122(5):882–7. 25704321 10.1016/j.ophtha.2015.01.013

[23] Bowman RJC, Kabiru J, Negretti G, Wood ML. Outcomes of bilateral cataract surgery in Tanzanian children. Ophthalmology. 2007;114(12):2287–92. 17448539 10.1016/j.ophtha.2007.01.030

[24] Lambert SR, Lynn MJ, Reeves R, Plager DA, Buckley EG, Wilson ME. Is there a latent period for the surgical treatment of children with dense bilateral congenital cataracts? J AAPOS. 2006;10(1):30–6. 16527677 10.1016/j.jaapos.2005.10.002

[25] Asferaw M, Mekonen SY, Woodruff G, Gilbert CE, Tesfaye S. Outcome of paediatric cataract surgery in Northwest Ethiopia: a retrospective case series. Br J Ophthalmol. 2019;103(1):112–8. 29669781 10.1136/bjophthalmol-2017-311513

[26] World Health Organization. International Statistical Classification of Diseases and Related Health Problems 10th revision Current Version for 2003 Chapter VII H54 Blindness and low vision. Available from: URL: https://www.who.int/classifications/classification-of-diseases.

[27] Rubin DB. Multiple Imputation for nonresponse in surveys. New York, NY: Wiley; 1987. (Wiley series in probability and mathematical statistics Applied probability and statistics). Available from: http://www.loc.gov/catdir/description/wiley034/86028935.html.

[28] Bronsard A, Geneau R, Shirima S, Courtright P, Mwende J. Why are children brought late for cataract surgery? Qualitative findings from Tanzania. Ophthal Epidemiol. 2008;15(6):383–8. 10.1080/09286580802488624 19065431

[29] Ebeigbe JA. Factors influencing eye-care seeking behaviour of parents for their children in Nigeria. Clin Exp Optom. 2018;101(4):560–4. 27990681 10.1111/cxo.12506

[30] Frech S, Hopkins A, Moanda A, Kilangalanga J, Guthoff RF. Social, educational and medical aspects after cataract surgery of bilaterally blind children in kinshasa-perception of parents and children. Children (Basel). 2022;9(11):1683. 36360411 10.3390/children9111683 PMC9688321

[31] The World Bank. Available from: https://www.worldbank.org/en/country/drc/overview.

[32] Ye H, Deng D, Qian Y, Lin Z, Chen W. Long-term visual outcome of dense bilateral congenital cataract. Chin Med J (Engl). 2007;120(17):1494–7. 17908457

