# Supplementary material for: Predictors of attendance at the first follow-up and poor visual outcome after paediatric cataract surgery in Kinshasa for the years 2001–2021
Source: Trop Med Health. 2025 Feb 26;53:32. doi: 10.1186/s41182-025-00706-8 (PMC11863572; doi:10.1186/s41182-025-00706-8)
Supplement: Supplementary file 1 — Supplementary Material 1: Table S1: Missing Data Pattern. Figure S1: Convergence of means Source: Saint Joseph Hospital (2001–2021). Table S2: Odds ratio (OR) of visual acuity in the first follow-up, multiple imputation. [file 41182_2025_706_MOESM1_ESM.docx]

Supplemental Material

**Table S1: Missing Data Pattern**

|  | **Pattern** | | | |
| --- | --- | --- | --- | --- |
|  | Visual acuity | Financial | Visual acuity | Visual acuity |
| **Percent** | (preoperative) | situation | (after surgery) | (Follow-up) |
| 53 | 1 | 1 | 1 | 1 |
| 27 | 1 | 1 | 1 | 0 |
| 9 | 1 | 1 | 0 | 0 |
| 9 | 1 | 1 | 0 | 1 |
| <1 | 1 | 0 | 1 | 1 |
| <1 | 1 | 0 | 0 | 1 |
| <1 | 0 | 1 | 0 | 1 |
| <1 | 0 | 1 | 0 | 0 |
| <1 | 0 | 1 | 1 | 1 |
| <1 | 1 | 0 | 1 | 0 |
| <1 | 0 | 0 | 0 | 1 |
| <1 | 0 | 1 | 1 | 0 |
| <1 | 1 | 0 | 0 | 0 |
| 100 |  |  |  |  |
| Source: Saint Joseph Hospital 2001 – 2021 | | | | |

53% of the observations had no missing values, 27% were missing in the dependent variable, 9% missing in the dependent variable and in visual acuity after operation, 9% in visual acuity after operation.

**Figure S1: Convergence of means**
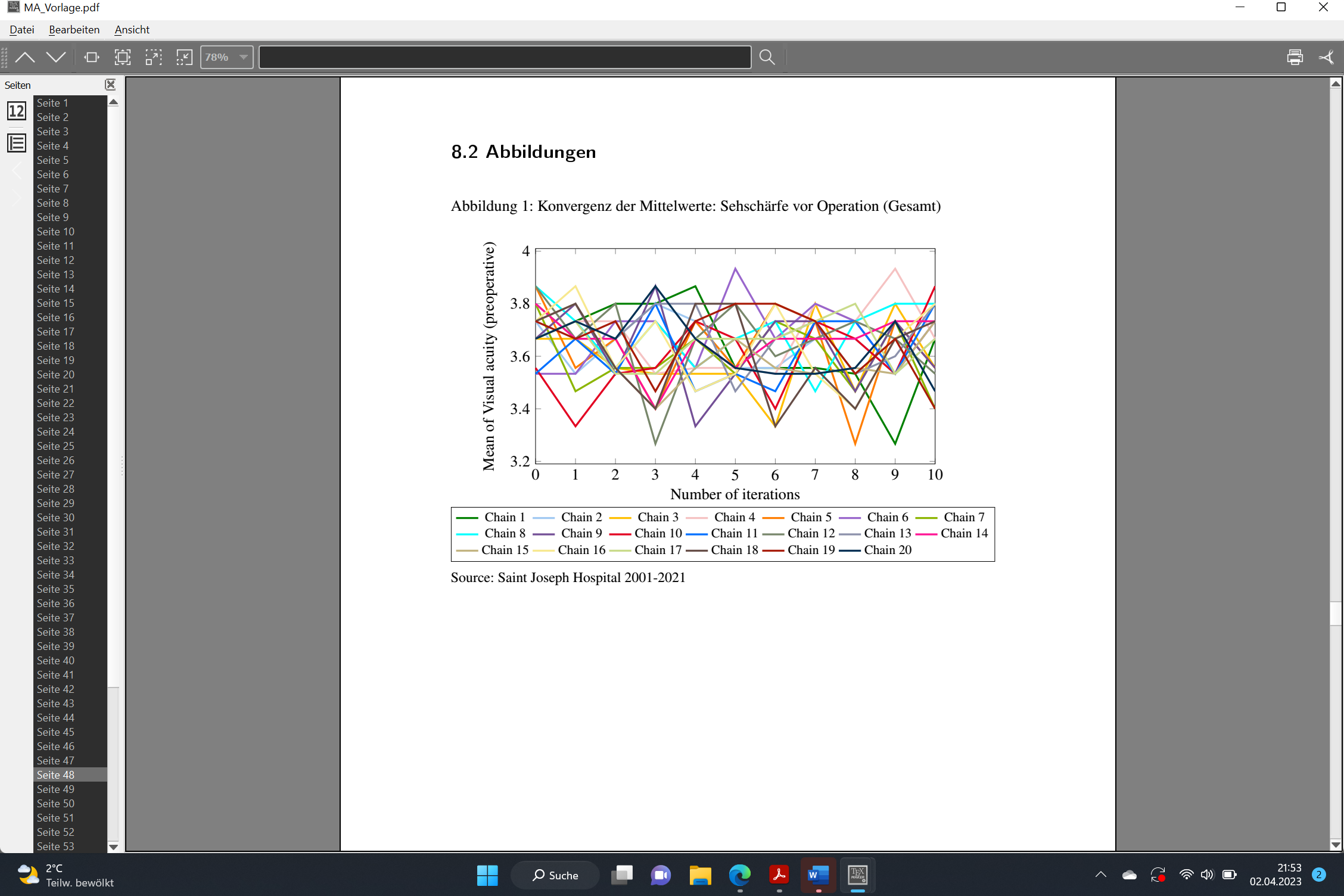
Source: Saint Joseph Hospital (2001 – 2021)


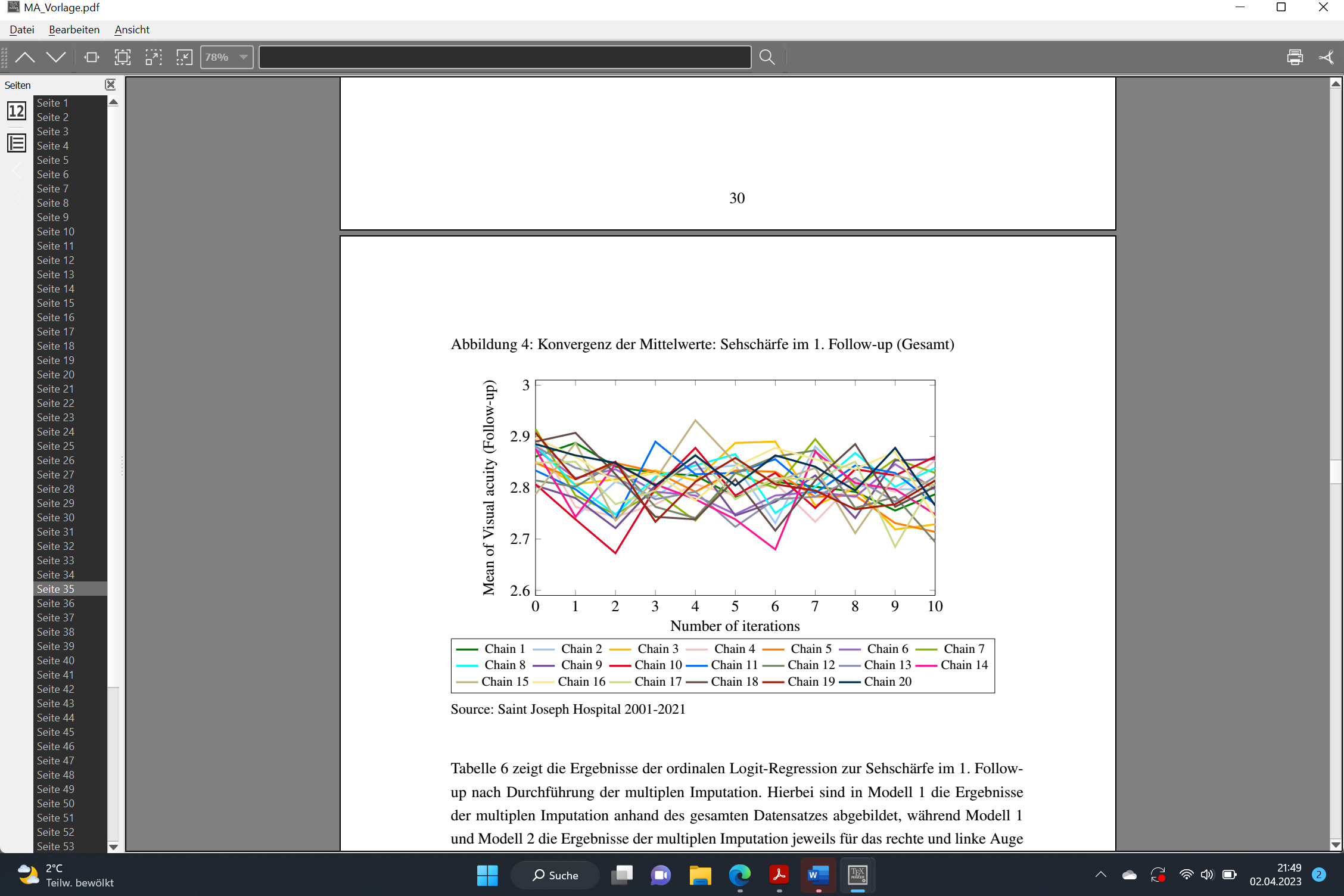
Source: Saint Joseph Hospital (2001 – 2021)


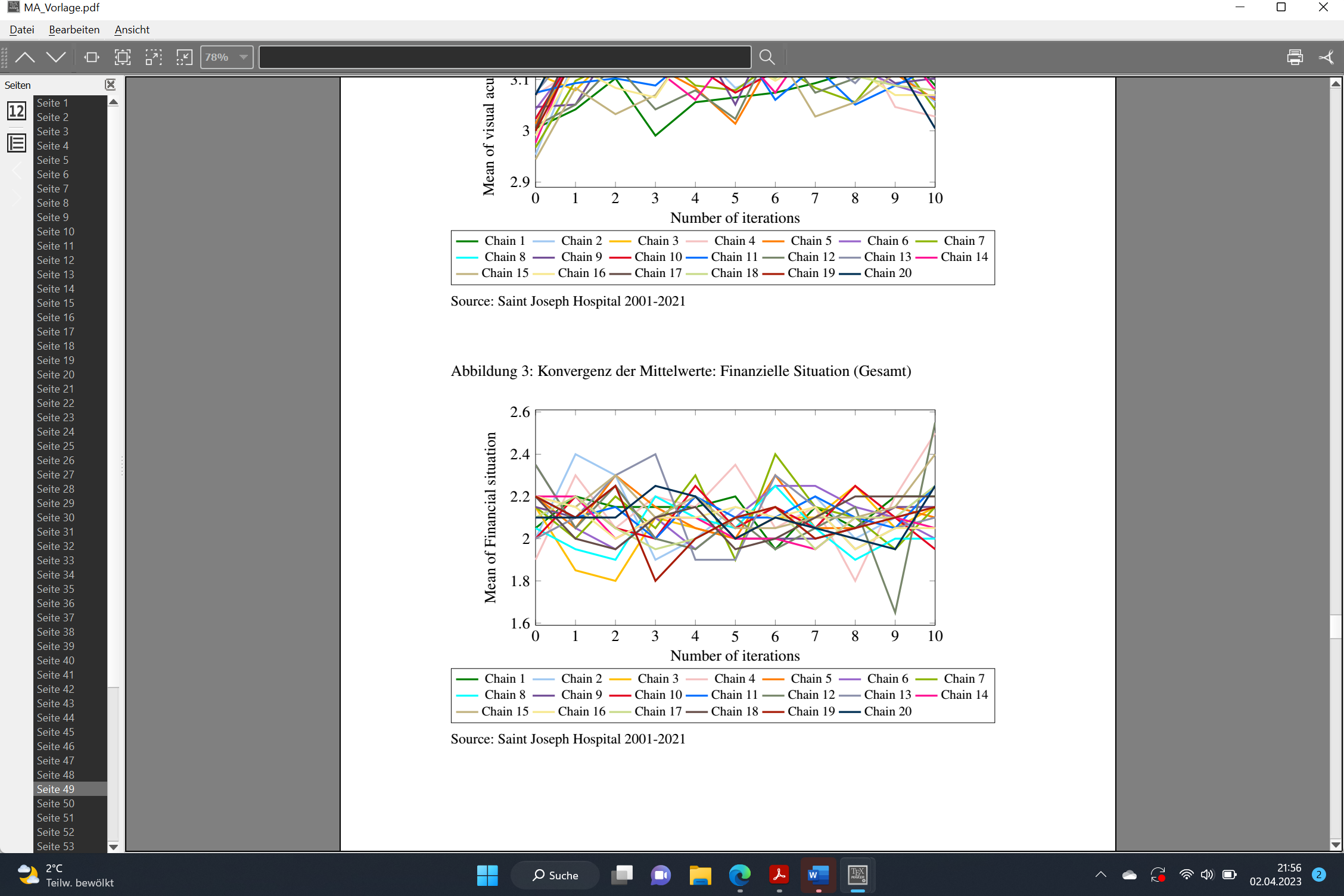
Source: Saint Joseph Hospital (2001 – 2021)


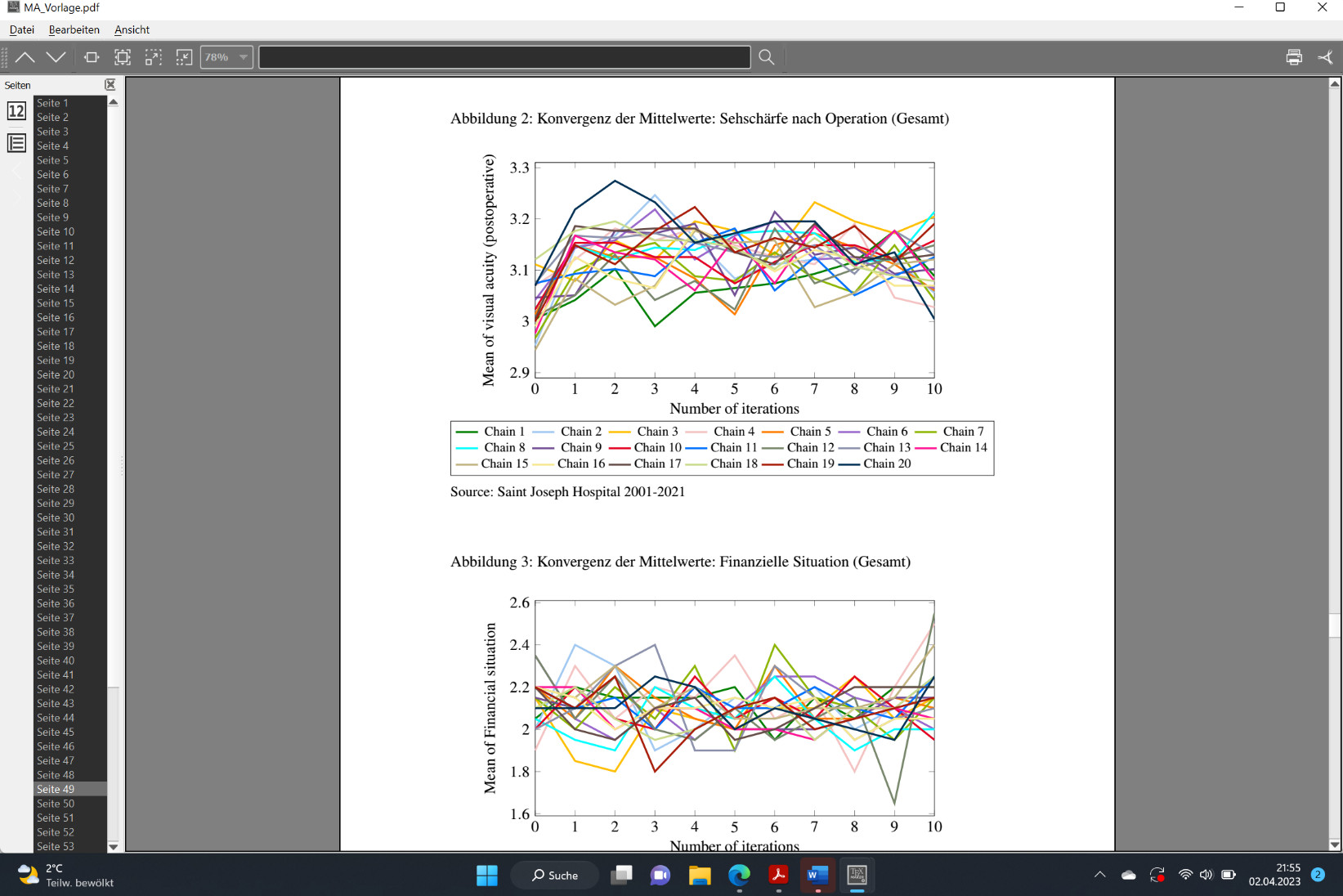


Source: Saint Joseph Hospital (2001 – 2021)

**Table S2: Odds ratio (OR) of visual acuity in the first follow-up, multiple imputation**

| Variable | Value | OR | p-value |
| --- | --- | --- | --- |
| Sex | Male | 1 |  |
|  | Female | 1.52 | 0.008 |
| Age | centered on median | 0.91 | <0.001 |
| Financial situation | Destitute/ Poor | 3.91 | 0.020 |
|  | Low income | 1 |  |
|  | Regular income | 1.13 | 0.479 |
| Visual acuity | Visual impairment | 1 |  |
| (preoperative) | Severe visual impairment | 1.16 | 0.668 |
|  | Blind/ Follow light or object | 2.15 | 0.027 |
| Visual acuity | Not impaired | 0.18 | 0.038 |
| (after surgery) | Visual impairment | 1 |  |
|  | Severe visual impairment/Blind/ Follow light or object | 3.49 | <0.001 |
| Laterality | Unilateral | 1 |  |
|  | Bilateral | 0.97 | 0.865 |
| Nystagmus | No | 1 |  |
| (preoperative) | Yes | 2.01 | <0.001 |
| Cut 1 | | 0.72 | |
| Cut 2 | | 2.96 | |
| Cut 3 | | 4.96 | |
| Cut 4 | | 6.51 | |
| N | | 1100 | |
| Source: Saint Joseph Hospital 2001 - 2021 | |  | |
|  |  |  |  |
|  |  |  |  |

Literature Cited

1. Bourne R, Steinmetz JD, Flaxman S, Briant PS, Taylor HR, Resnikoff Sea. Trends in prevalence of blindness and distance and near vision impairment over 30 years: an analysis for the Global Burden of Disease Study. Lancet Glob Health 2021; 9(2):e130-e143.

2. Solebo AL, Teoh L, Rahi J. Epidemiology of blindness in children. Arch Dis Child 2017; 102(9):853–7.

3. Naipal S, Rampersad N. A review of visual impairment. Afr. vis. eye health 2018; 77(1).

4. Gogate P, Gilbert C. Blindness in children: a worldwide perspective. Community Eye Health 2007; 20(62):32–3.

5. Amiebenomo OM, Achugwo DC, Abah I. Parental knowledge and attitude to children's eye care services. Nig. J. Paed. 2016; 43(3):215.

6. Welp A, Woodbury RB, McCoy MA, Teutsch SM, editors. Making Eye Health a Population Health Imperative: Vision for Tomorrow. Washington (DC); 2016.

7. World Health Organization. World report on vision. Geneva: WHO; 2019.

8. Kilangalanga JN, Stahnke T, Moanda A, Makwanga E, Hopkins A, Guthoff RF. Role of a Community-based Program for Identification and Referral of Pediatric Cataract Patients in Kinshasa, Democratic Republic of the Congo. Middle East Afr J Ophthalmol 2019; 26(2):83–8.

9. Yorston D, Wood M, Foster A. Results of cataract surgery in young children in east Africa. Br J Ophthalmol 2001; 85(3):267–71.

10. Limburg H, Foster A, Gilbert C, Johnson GJ, Kyndt M, Myatt M. Routine monitoring of visual outcome of cataract surgery. Part 2: Results from eight study centres. Br J Ophthalmol 2005; 89(1):50–2.

11. Eriksen JR, Bronsard A, Mosha M, Carmichael D, Hall A, Courtright P. Predictors of poor follow-up in children that had cataract surgery. Ophthalmic Epidemiol 2006; 13(4):237–43.

12. Rai SKC, Thapa H, Kandel RP, Ishaq M, Bassett K. Clinical and cost impact of a pediatric cataract follow-up program in western Nepal and adjacent northern Indian States. J AAPOS 2014; 18(1):67–70.

13. Gogate P, Patil S, Kulkarni A, Mahadik A, Tamboli R, Mane R et al. Barriers to follow-up for pediatric cataract surgery in Maharashtra, India: how regular follow-up is important for good outcome. The Miraj Pediatric Cataract Study II. Indian J Ophthalmol 2014; 62(3):327–32.

14. Mndeme FG, Mmbaga BT, Msina M, Mwende J, Vaitha SJ, Kim MJ et al. Presentation, surgery and 1-year outcomes of childhood cataract surgery in Tanzania. Br J Ophthalmol 2021; 105(3):334–40.

15. Congdon NG, Ruiz S, Suzuki M, Herrera V. Determinants of pediatric cataract program outcomes and follow-up in a large series in Mexico. J Cataract Refract Surg 2007; 33(10):1775–80.

16. Huang G, Crooms R, Chen Q, Congdon N, He M. Compliance with follow-up after cataract surgery in rural China. Ophthalmic Epidemiol 2012; 19(2):67–73.

17. Bright T, Felix L, Kuper H, Polack S. Systematic review of strategies to increase access to health services among children over five in low- and middle-income countries. Trop Med Int Health 2018; 23(5):476–507.

18. Adams C, Alex AA, Trivedi RH, Wilson ME. Outcomes of bilateral cataract surgery in children 2-7 years of age: a comparison to surgery in toddlers and infants. J AAPOS 2022; 26(3):133.e1-133.e6.

19. Chan WH, Biswas S, Ashworth JL, Lloyd IC. Congenital and infantile cataract: aetiology and management. Eur J Pediatr 2012; 171(4):625–30.

20. Tomkins O, Ben-Zion I, Moore DB, Helveston EE. Outcomes of pediatric cataract surgery at a tertiary care center in rural southern Ethiopia. Arch Ophthalmol 2011; 129(10):1293–7.

21. Mailu EW, Virendrakumar B, Bechange S, Jolley E, Schmidt E. Factors associated with the uptake of cataract surgery and interventions to improve uptake in low- and middle-income countries: A systematic review. PLoS One 2020; 15(7):e0235699.

22. Negretti GS, Ayoub T, Ahmed S, Deb R, Majumder U, Jewel J et al. Cataract surgery outcomes in bangladeshi children. Ophthalmology 2015; 122(5):882–7.

23. Bowman RJC, Kabiru J, Negretti G, Wood ML. Outcomes of bilateral cataract surgery in Tanzanian children. Ophthalmology 2007; 114(12):2287–92.

24. Lambert SR, Lynn MJ, Reeves R, Plager DA, Buckley EG, Wilson ME. Is there a latent period for the surgical treatment of children with dense bilateral congenital cataracts? J AAPOS 2006; 10(1):30–6.

25. Asferaw M, Mekonen SY, Woodruff G, Gilbert CE, Tesfaye S. Outcome of paediatric cataract surgery in Northwest Ethiopia: a retrospective case series. Br J Ophthalmol 2019; 103(1):112–8.

26. World Health Organization. International Statistical Classification of Diseases and Related Health Problems 10th revision Current Version for 2003 Chapter VII H54 Blindness and low vision. [cited 2023 Jun 6]. Available from: URL: https://www.who.int/classifications/classification-of-diseases.

27. Rubin DB. Multiple Imputation for nonresponse in surveys. New York, NY: Wiley; 1987. (Wiley series in probability and mathematical statistics Applied probability and statistics). Available from: URL: http://www.loc.gov/catdir/description/wiley034/86028935.html.

28. Bronsard A, Geneau R, Shirima S, Courtright P, Mwende J. Why are children brought late for cataract surgery? Qualitative findings from Tanzania. Ophthalmic Epidemiol 2008; 15(6):383–8.

29. Ebeigbe JA. Factors influencing eye-care seeking behaviour of parents for their children in Nigeria. Clin Exp Optom 2018; 101(4):560–4.

30. Frech S, Hopkins A, Moanda A, Kilangalanga J, Guthoff RF. Social, Educational and Medical Aspects after Cataract Surgery of Bilaterally Blind Children in Kinshasa-Perception of Parents and Children. Children (Basel) 2022; 9(11).

31. The World Bank [cited 2024 Jan 11]. Available from: URL: https://www.worldbank.org/en/country/drc/overview.

32. Ye H, Deng D, Qian Y, Lin Z, Chen W. Long-term visual outcome of dense bilateral congenital cataract. Chin Med J (Engl) 2007; 120(17):1494–7.
